# Controversial Outcomes in Neck Rehabilitation between Surgically and Conservatively Treated Patients—Results of an Observational Study

**DOI:** 10.3390/jcm12031004

**Published:** 2023-01-28

**Authors:** Martin Missmann, Vincent Grote, David Riedl, Jean-Pascal Grenier, Michael J. Fischer

**Affiliations:** 1 Austrian Workers’ Compensation Board AUVA, Ingenieur-Etzel-Str. 17, 6020 Innsbruck, Austria; 2 Ludwig Boltzmann Institute for Rehabilitation Research, Kurbadstraße 14, 1100 Vienna, Austria; 3 Vamed Rehabilitation Center Kitzbühel, Hornweg 32, 6370 Kitzbühel, Austria; 4 Hannover Medical School MHH, Clinic for Rehabilitation Medicine, Carl-Neuberg-Straße 1, 30625 Hannover, Germany

**Keywords:** rehabilitation, cervical spine, neck pain, surgical treatment, conservative treatment, outcomes, critical success factors, performance score

## Abstract

The present study aimed to compare changes during inpatient rehabilitation between conservatively and surgically treated patients. A total of *n* = 162 patients with cervical spine complaints were included in the study (*n* = 107 conservatively treated, *n* = 55 after surgery). Patients completed disease-specific (NDI) and generic (NPRS, EQ-5D-5L, HAQ) patient reported outcome measures (PROMs) before and after rehabilitation. In addition, the range of motion (ROM) in the transversal plane of the cervical spine was measured. Changes and correlations between PROMs and ROM values during rehabilitation were assessed. The influence of moderating factors on NDI outcomes was examined. Significant improvements with large effect sizes were found in PROMs and ROM (all *p* < 0.001). The conservatively treated patients showed significantly greater NDI improvements than operated patients (*p* = 0.050), but a greater proportion of poor performance in ROM (*p* = 0.035). Baseline NDI (β = 0.66), HAQ (β = 0.14), and ROM scores (β = −0.17) explained 63.7% of the variance in NDI after rehabilitation. Both patient groups showed different outcomes. The findings of this study indicate that the unique needs of patients may require different therapeutic interventions and highlight the importance of using multidimensional outcome measures when implementing a multimodal rehabilitation approach.

## 1. Introduction

Pain and impairment of the cervical spine are common orthopaedic disorders with a lifetime incidence of approximately 22% to 70% of the population and an annual prevalence of 30% to 50% [1,2]. Female gender, older age, high job demands, being an ex-smoker, having low social or work support, and having a previous history of neck or low back disorders were identified as risk factors for developing neck pain [3]. Conditions of the cervical spine are related to traumatic or degenerative alterations, but may also be caused by internal or neurological pathologies that have to be excluded during the initial clinical examination [4].

The human cervical spine normally consists of seven vertebrae, providing attachment points for muscles that support and move the upper limb girdle and that suspend and move the inlet of the thorax [5]. The vertebrae are separated from one another by intervertebral discs; these enable movement of the cervical spine and the head together with the vertebral joints. The vertebral arches and bodies enclose the spinal cord, while the spaces between the vertebrae serve as corridors for the paired spinal nerves. Ligaments of the cervical spine include the alar ligaments, anterior and posterior longitudinal ligament, and ligamenta flava. Skeletal muscles attached to the cervical spine, such as the sternocleidomastoid, trapezius, levator scapulae, erector spinae, deep cervical flexors, and suboccipital muscles, enable movements of the head and neck and, in part, of the shoulder girdle.

Tumours, trauma, inflammatory diseases, and neurological disorders may restrict head and neck mobility. In addition, the cervical spine undergoes progressive, age-related, non-inflammatory degenerative changes [6] referred to as spondylosis [7,8] that can cause pain and lead to restricted mobility. Spondylosis is caused by local ossification and the formation of osteophytes and, taken together with thickening and buckling of the ligamentum flavum, is one of the reasons for myelopathy and nerve radiculopathy [9]. Cervical disc herniation (CDH) develops from the degenerative rupture of the annulus fibrosus and the protrusion of the nucleus pulposus, resulting in the compression and irritation of spinal nerves and other structures [10]. While cervical disc pathologies remain asymptomatic in some cases [11], CDH is a common cause of localized myelopathy and cervical radiculopathy [12]. As a result of these pathologies, patients may develop neck pain or stiffness, hyperreflexia, and motor weakness, as well as experience sensory changes in the local and corresponding caudal nerve segments [13].

According to Fritz and Brennan [14], neck pain is classified into four categories: (1) pain with mobility deficits, (2) pain with movement coordination impairments, (3) pain with headache, and (4) pain with radiating pain. Clarke et al. [2] proposed that a distinction should be made between axial and radicular pain, which typically extends distally down the upper extremity in a dermatomal distribution. Depending on this extension, they recommended conservative treatment for axial pain rather than for radicular pain. However, Engquist et al. [15] identified specific factors that led to a better outcome in certain patients after surgery as compared to patients who underwent conservative treatment.

Functional status and current subjective condition are determined from objective measurements by clinicians (‘clinician reported outcome measures’, CROMs) and by patient-reported outcome measures (PROMs). Although the value of measuring the range of motion (ROM) is not entirely clear, limited ROM of the cervical spine is still one of the most commonly assessed physical impairments in clinical practice, indicating that restricted cervical ROM might be associated with negative outcomes [16]. While generic PROMs provide information about the patient’s general health status [17], specific PROMs describe outcomes related to a particular injury or disease [18]. Both CROMs and PROMs may show characteristic changes during rehabilitation. The correlation between these methods typically lies between 0.2 and 0.6 [19,20,21]. The generic PROMs used in this study were the Health Assessment Questionnaire (HAQ) [22] and the European Quality of Life-5 Dimensions questionnaire (EQ-5D) [23], whereas the Neck Disability Index (NDI) [24] served as a specific PROM for describing limitations caused by neck pain.

This retrospective cohort study was carried out to evaluate the effects of multimodal rehabilitation in patients who had previously undergone either surgical or conservative treatment. For this reason, inpatients at a specialized rehabilitation facility for cervical spine disorders were divided into two groups, one treated conservatively and one treated surgically, to compare outcomes in terms of the ROM, HAQ, EQ-5D, Numeric Pain Rating Scale (NPRS), and NDI.

## 2. Materials and Methods

### 2.1. Procedure

Inpatients with complaints of the cervical spine at a specialized orthopaedic rehabilitation facility were consecutively included in this retrospective cohort study. Patients were assigned to two groups: (a) the conservative treatment group (‘cons-group’), comprised patients who suffered from local or radicular pain caused by a narrowing of the spinal canal or nerve root canal due to osteochondrosis, spinal disk protrusion, or herniation; and (b) the surgery group (‘op-group’), in which patients had previously undergone surgery as a result of compression of the spinal cord or nerve roots.

Both patient groups took part in an interdisciplinary inpatient rehabilitation treatment programme. This inpatient program lasted 21 days, as defined in the service portfolio of the Austrian social security institutions [25]. The medical treatments lasted an average of 2–3 h per day and amounted to at least 1800 min of therapy [26,27]. Patients routinely completed generic and disease-specific patient-reported outcome (PRO) questionnaires before (*T*1) and at the end of rehabilitation (*T*2). Prior to completing the questionnaires, patients were informed about the use of the data for research purposes, provided with a quality assurance document, and signed a written informed consent form.

### 2.2. Outcome Measures

#### 2.2.1. Range of Motion (ROM)

A number of tools have been designed to measure the joint range of motion, varying from simple visual estimation to high-speed cameras, using a conventional goniometer, digital devices, or a radiographic joint angle measurement [28,29]. As in the present study, the universal full circle goniometer is the preferred instrument for measuring the axial rotation of the cervical spine [30,31]. For statistic evaluation, we converted the degree values to a proportion of the normal axial range of motion.

#### 2.2.2. Numeric Pain Rating Scale (NPRS)

Pain has a major impact on physical, emotional, and cognitive functioning [32,33] and is assessed by patients themselves using a numeric pain rating scale (NPRS). The NPRS describes the intensity of pain on a 0–10 scale, with zero meaning “no pain”, and ten meaning “the worst pain imaginable” [34].

#### 2.2.3. Health Assessment Questionnaire (HAQ)

The HAQ is one of the most widely used comprehensive, validated, patient-oriented outcome assessment instruments [22,35]. The HAQ was originally developed in 1978 by James F. Fries and his colleagues at Stanford University and has been validated in patients with a wide variety of rheumatic diseases. The HAQ is composed of 20 items in the following eight categories: 1. Dressing and Grooming, 2. Arising 3. Eating, 4. Walking, 5. Hygiene, 6. Reach, 7. Grip, and 8. Common Daily Activities. For each of the categories, patients report the amount of difficulty they have when performing the two or three specific sub-category items, using four possible responses for each item or component: 0 = without any difficulty, 1 = with some difficulty, 2 = with much difficulty, and 3 = unable to do.

#### 2.2.4. EuroQol—5 Dimensions—5 Levels (EQ-5D-5L)

The EQ-5D-5L is a generic instrument that is used to measure five dimensions of the health status, and each dimension is comprised of five levels: mobility, self-care, daily activities, pain/discomfort, and anxiety/depression [23]. In addition to a five-item descriptive system, this instrument contains a vertical visual analogue scale (VAS) ranging from 0 (‘the best health you can imagine’) to 100 (‘the worst health you can imagine’), which the patient uses to record (i.e., self-rated) their health [36,37]. The five levels range from level 1 (no problems) to level 5 (extreme problems/unable to do) [38].

#### 2.2.5. Neck Disability Index (NDI)

The NDI is a specific PROM and was developed by Vernon and Mior in 1991 [24]. This index was originally based on the Oswestry Low Back Pain Questionnaire by Fairbank, Couper, and Davies [39] and consists of ten sections based on the intensity of the pain: (1), personal care (2), lifting (3), work (4), headaches (5), concentration (6), sleeping (7), driving (8), reading (9), and concentration (10). Six answers are possible for each of these sections, yielding a score of 0–5 points. The sum of the scores obtained is doubled to provide a percentage score (0–20, normal; 21–40, mild disability; 41–60, moderate; 61–80, severe; and 80+, complete/exaggerated) [40].

### 2.3. Statistical Analyses

**Figure 1:**
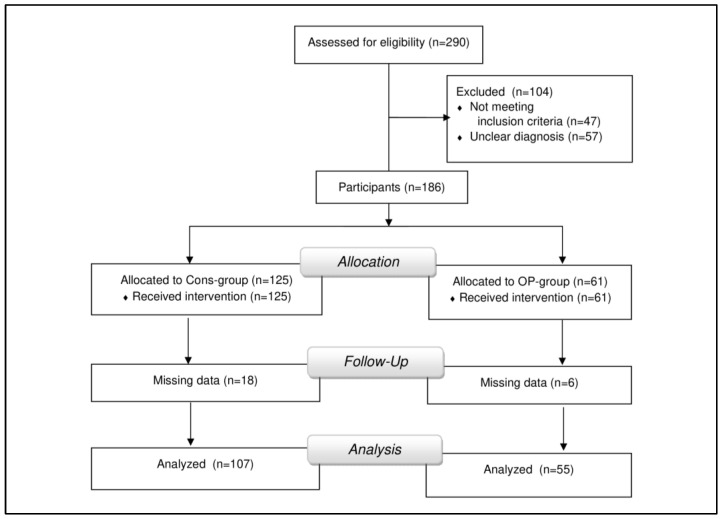
Flow chart illustrating the participant recruitment procedure.

The sample of participants was divided into two groups of patients who had previously undergone surgery (*n* = 55; op-group) or conservative treatment (*n* = 107; cons-group; Figure 1). Descriptive analyses were given for pain (NPRS), generic PROMs (HAQ, EQ-5D-5L), neck-specific PROMs (NDI), and Range of Motion (ROM) in either group. Baseline group differences were investigated with independent sample *t*-tests and χ^2^ tests. We analyzed associations of PROMs and ROM by calculating the Pearson correlation coefficients. Associations between baseline PROM and ROM scores, as well as between change scores (delta: *T*2 − *T*1), were analyzed. Because patients in the cons-group and op-group displayed significant differences in ROM scores, we also tested whether using the “performance score (T2D)” could improve the comparability of PROMs and ROM.

The T2D is a simple method to correct for baseline differences when comparing group outcomes. It can be calculated with the formula *T*2 + (*T*2 − *T*1), which reflects the individual performance and considers the functional status at the beginning of rehabilitation (changes from *T*1 to *T*2; Δ) [20,21,41,42,43], without having to deal with problems of mathematical coupling or regression effects as seen in ANCOVA. In addition to performing interference statistics, we also calculated a scatter plot to visualize correlations in performance (T2D scores) between NDI and ROM.

Changes in PROMs during rehabilitation were investigated by calculating repeated measures analyses of variance (rANOVA). To investigate differences in terms of changes that occurred during rehabilitation between cons-group and op-group patients, the grouping variable was added to the rANOVA. The magnitude of mean differences were evaluated using η^2^ effect sizes [41], with values of η^2^ = 0.01 considered as small, η^2^ = 0.06 as medium, and η^2^ = 0.14 as large effect sizes, respectively [44]. In addition, we grouped patients into categories of good, average, and bad performers based on their NDI T2D tertile scores and compared the representation of these groups in the cons-group and op-group using a χ^2^-test.

To determine which characteristics were associated with the NDI score after rehabilitation treatment (T2) (i.e., ‘critical success factors’), hierarchical multiple linear regression analyses were calculated in four steps by successively adding the following variables: (1) sociodemographic variables (age, sex, BMI), (2) treatment intensity data (number of psychological, physiotherapeutic and occupational therapy treatments, and number of medical counselling), (3) PROM scores (EQ-5D-5L total score, HAQ, NPRS) and ROM at baseline (*T*1), and (4) the NDI baseline score. The goodness of fit was determined by calculating Durbin-Watson statistics (values between 1.5–2.5 were deemed acceptable) and VIF (values < 10.0 were deemed acceptable). Calculations were performed for the total sample first and then repeated for the cons-group and the op-group separately.

All calculations were performed using IBM SPSS (v.21). Values of *p* < 0.05 were considered to be statistically significant.

## 3. Results

Initially, a total of *n* = 290 inpatients were assessed for eligibility (Figure 1). Of these, *n* = 47 who had complaints predominantly of the lumbar spine, suffered from somatoform or psycho-vegetative disorders, experienced severe trauma to the cervical spine, or had undergone cervical spine surgery more than one year previously and were thus excluded from the study. Another *n* = 57 patients were excluded because they could not localize their pain and reported diffuse spinal complaints either without providing further information or that arose due to a rheumatic disease or to spinal deformity in scoliosis. Finally, *n* = 24 patients were excluded due to missing PROMs or ROM data. The remaining *n* = 162 patients were included in the study. The recruitment procedure is displayed in Figure 1.

### 3.1. Patient Characteristics

Most patients were female (60.5% vs. 39.5%) and had a mean age of 52.7 (SD: 8.6) years (Table 1). Most patients were obese (BMI > 25; *n* = 96, 59.3%) but only one patient was underweight (BMI < 18.5; *n* = 1; 0.6%).

### 3.2. Rehabilitation Treatment

Members of both groups underwent a 21-day in-rehabilitation program, including 100.80 ± 41.79 min (range: 65–265) of individual doctor consultation and, for all participants, individual physiotherapy lasting 236.05 ± 58.56 min (range: 150–420) thermo- and electrotherapy, and massage therapy lasting 182.57 ± 23.97 min (range: 150–360).

Only a minor number of patients selected additional therapies: *n* = 47 chose occupational therapy (107.23 ± 47.12 min, range 30–270), *n* = 55 consulted a dietician (71.54 ± 28.83 min, range 30–150), *n* = 16 took part in a pain therapy group (63.75 ± 15.00 min, range 60–120), and *n* = 55 patients consulted a psychologist (88.90 ± 38.71 min, range 30–210). Some of the patients were both in the pain therapy group and/or visited a psychologist. The content of these two therapy forms was comparable; thus, the two therapies were merged to evaluate the results, with 60 patients in one and/or the other group (98.5 ± 42.46 min, range 30–210).

### 3.3. Association of PROMs and ROM

At baseline, all assessed PROMs were significantly associated with each other. The strongest associations were found between higher EQ-5D-5L impairment scores and higher NDI scores (*r* = 0.74, *p* < 0.001) and higher HAQ scores (*r* = 0.67, *p* < 0.001). However, the ROM scores were not significantly associated with any of the PROM scores at baseline. For detailed information, see Table 2.

As shown in Table 3, the changes in the NDI between *T*1 and *T*2 were significantly associated with changes in the NPRS score (*r* = 0.31, *p* < 0.01), EQ-5D-5L score (*r* = 0.37, *p* < 0.001), and HAQ score (*r* = 0.24, *p* < 0.05), and changes in the EQ-5D-5L were also associated with the HAQ score (*r* = 0.63, *p* < 0.001). However, changes in the ROM were not associated with changes in any of the PROMs.

**Figure 2:**
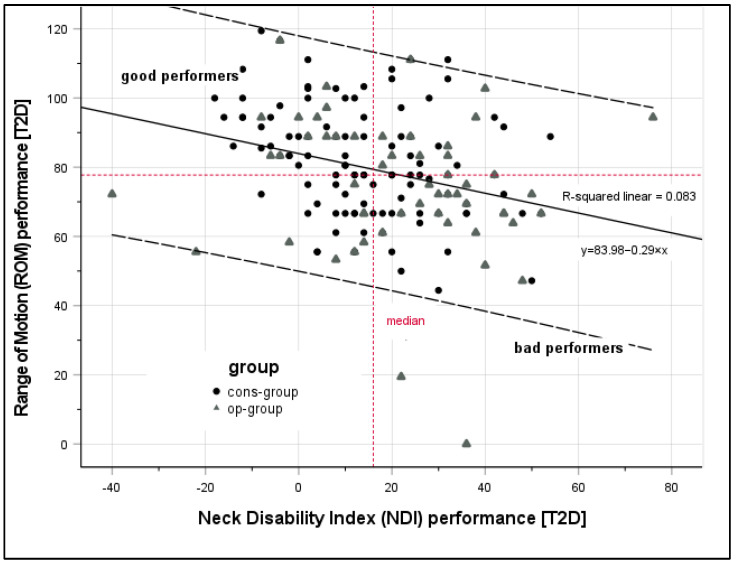
Scatter plot of T2D scores [*T*2 + (*T*2 − *T*1)] of Range of Motion (ROM) and Neck Disability Index (NDI).

In a next step, we tested if the use of the T2D scores improved associations of changes in ROM and PROMs. In support of our hypothesis, after T2D correction, increased ROM scores were associated with decreased NDI (*r* = −0.33, *p* < 0.001) and decreased EQ-5D-5L scores (*r* = 0.26, *p* < 0.01). For detailed information, see Table 4. This trend could also be visualized when comparing scatterplots of ROM_T2D_ and NDI_T2D_. As shown in Figure 2, the performance score provides a more coherent picture than the change values (Table 3) when the T2D algorithm is applied, regardless of group assignment.

### 3.4. Mean Changes during Rehabilitation Treatment

Patients showed statistically significant amounts of improvement over the course of rehabilitation (Table 5) with large effect sizes for NPRS (*p* < 0.001), EQ-5D-5L total score (*p* < 0.001), NDI (*p* < 0.001), and ROM scores (*p* < 0.001). Only for the HAQ score could no significant improvement be observed during the rehabilitation (*p* = 0.57).

**Figure 3:**
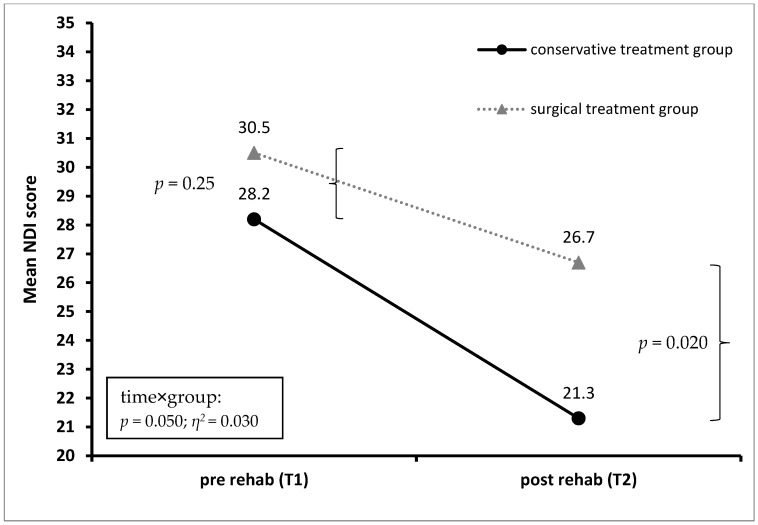
Mean Neck Disability Score (NDI) before (*T*1) and after (*T*2) rehabilitation treatment for patients in the conservative and the surgical treatment group.

**Figure 4:**
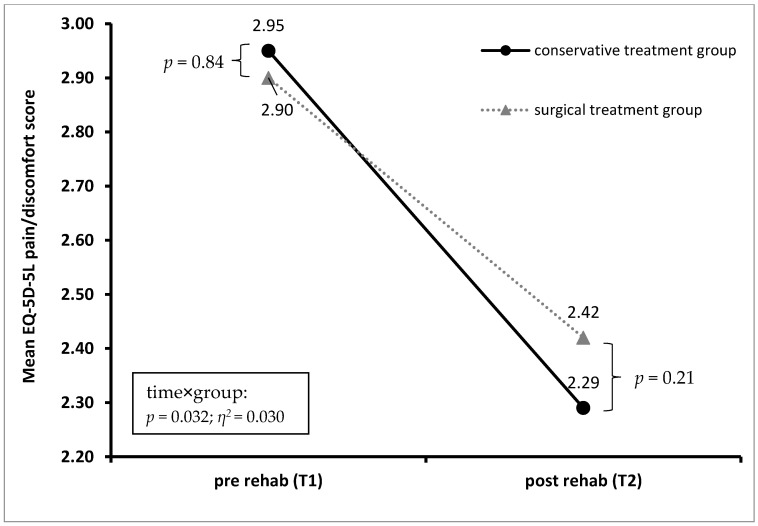
Mean EQ-5D-5L pain/discomfort score before (*T*1) and after (*T*2) rehabilitation treatment for patients in the conservative and surgical treatment groups.

Patients in the cons-group showed a statistically significant larger amount of improvement in terms of the NDI score (*p* = 0.050) and the EQ-5D-5L pain/discomfort score (*p* = 0.032) than did patients in the op-group, with a small effect size. For detailed information, see Figure 3 and Figure 4.

Regarding the NPRS, EQ-5D-5L total score, and the ROM score, patients in the conservative treatment group showed larger amounts of improvement, but these differences were not statistically significant (*p* = 0.61–0.12). For detailed information, see Table 5.

**Figure 5:**
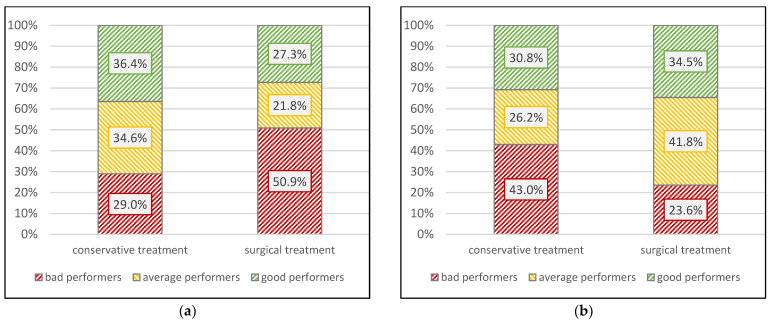
Distribution of good, average, and bad performers (NDI and ROM T2D tertiles) stratified by group. (**a**) Group Neck Disability Index (NDI) performance; (**b**) Group performance in Range of Motion (ROM).

When patients were grouped into categories of good, average, and bad performers based on the T2D scores of the NDI, a statistically significant difference was detected between the two treatment groups (χ^2^ = 7.67, *p* = 0.022), with patients in the op-group showing a larger proportion of bad performers (50.9% vs. 29.0%) and fewer average (21.8% vs. 34.6%) and fewer good performers (27.3% vs. 36.4%). Regarding the performance on the basis of the T2D ROM scores, a statistically significant inversed effect was observed (χ^2^ = 6.72, *p* = 0.035): patients in the cons-group showed more bad performers (43.0% vs. 23.6%) and fewer average (26.2% vs. 41.8%) and fewer good performers (30.8% vs. 34.5%) (Figure 5).

### 3.5. Critical Success Factors for Neck Disability Score at the End of Treatment (T2)

To determine which factors were associated with the NDI score at the end of inpatient rehabilitation, a hierarchical multiple linear regression model was designed. The model consisted of three steps, consecutively including sociodemographic and health-behaviour factors, baseline PROM scores, and treatment-related factors (Table 6). Model statistics indicated no auto-correlation (Durbin-Watson = 1.97) or multicollinearity (VIF = 1.12–2.80), and the final model explained 63.4% of the variance of the NDI score at *T*2 (*p* < 0.001).

Of the five sociodemographic and health-behaviour variables added in step 1, having a female sex (β = 0.18; *p* = 0.019) and higher number of medications (β = 0.35; *p* < 0.001) were associated with a higher NDI score at *T*2. After including the treatment factors in step 2, sex was no longer a significant predictor. Patients with higher NDI scores at *T*2 took part significantly more often in psychological treatment (β = 0.24; *p* = 0.003) and medical counselling (β = 0.21; *p* = 0.005). The explained variance significantly increased to 28.4% after including the variables in step 2 (*p* = 0.001). However, when baseline PROMs were added to the model in step 3, only the higher frequency of medical counselling (β = 0.14; *p* = 0.039) remained as a statistically significant predictor. The overall explained variance significantly increased to 48.0% (*p* < 0.001), and higher baseline HAQ (β = 0.31; *p* < 0.001) scores as well as lower ROM scores (β = −0.18; *p* = 0.008) were associated with higher NDI scores at the end of treatment. Finally, when adding the NDI baseline score (*T*1) to the model, medical counselling no longer served as a significant predictor. Higher baseline NDI (β = 0.66; *p* < 0.001) and HAQ (β = 0.14; *p* = 0.047) scores as well as lower ROM scores (β = −0.17; *p* = 0.003) explained 63.7% of the NDI score at the end of rehabilitation (*p* < 0.001). For detailed information, see Table 6.

The analysis was repeated for both treatment groups separately. While the overall results remained comparable, slight differences could be observed: The number of medications was the only significant predictor in step 1 for both patients in the cons-group (β = 0.27; *p* = 0.007) and the op-group (β = 0.34; *p* = 0.013). In step 2, only the number of psychological treatments was associated with higher NDI scores at the end of rehabilitation (β = 0.33; *p* = 0.002) in the cons-group, while the number of medical counselling was the only significant predictor in the op-group (β = 0.32; *p* = 0.028). In step 3, higher EQ5D-5L scores (β = −0.38; *p* = 0.004) and lower ROM (β = −0.16; *p* = 0.049) scores were significant predictors in the cons-group, but higher HAQ scores (β = 0.43; *p* = 0.008) were the sole predictor in the op-group. In step 4, higher NDI (β = 0.64; *p* < 0.001) and lower ROM (β = −0.17; *p* = 0.012) were significant predictors in the cons-group, while in the op-group, only the NDI (β = 0.72; *p* < 0.001) was a significant predictor.

## 4. Discussion

The aim of the present study was to investigate if patients who had previously undergone either surgical treatment or conservative treatment profit differently from multi-modal rehabilitation treatment.

While we observed significant improvements in the anxiety/depression and pain/discomfort scores in both treatment groups, patients in the cons-group showed a tendency to display larger amounts of improvement. These findings are similar to those of Cha et al. [45], who found significantly worse NDI scores in depressive patients at certain time points after surgery than in non-depressive patients. However, not all patients who required surgical treatment were depressed. This may also explain the small group differences for the EQ-5D values seen in our cohort, which contrast with those seen in a study on preoperative mental health in patients undergoing cervical spine surgery [46]. The cons-group showed comparable results, but a higher correlation between pain and NDI at *T*2 (*r* = 0.69, *p* = 0.004) was observed as compared to the op-group (*r* = 0.32, *p* = 0.014). In addition, no correlation was detected between the NDI at *T*1 and ROM at *T*2 (*r* = 0.07, *p* = 0.46).

As seen in previous research [47], a high disability (NDI) was a significant predictor of rehabilitation outcome after a three-week rehabilitation program in both the conservative and the surgical group in our study. Additionally, we found that psychological treatment was associated with rehabilitation outcome in the cons-group, while medical counselling was the treatment-related predictor in the op-group.

Giesinger et al. [48] detected different outcomes for generic and specific PROMs in knee rehabilitation and demonstrated that outcome measures differed significantly in terms of their responsiveness. Joint-specific PROMs were more responsive than clinician-reported or generic outcome measures. In contrast, we only found moderate correlations between specific and generic PROMs in neck rehabilitation. Both objective measures and PROMs may display characteristic changes during rehabilitation. The correlation between these methods typically lies between 0.2 and 0.6 [19,20,21]. Which outcome measure is the most appropriate to accurately capture the course and outcome of rehabilitation is debatable. In a cohort study of 4293 young male adults, Kauther et al. [49] examined the association between cervical ROM and neck pain. As we found, these authors found no correlation between ROM and neck pain; however, when controlling for baseline differences by using the T2D, changes in ROM and NDI scores were significantly associated with improvements in ROM linked to reduced neck disability. These results highlight the usefulness of T2D when comparing different outcome measures in orthopaedic rehabilitation research.

In another prospective cohort study, ROM and isometric neck muscle strength were assessed at baseline to determine whether the ROM of the cervical spine or neck muscle strength could predict the development of neck pain. Based on sixteen years of data, no association between neck muscle strength or ROM and the development of neck pain and disability was found [50]. It is worth noting that, with one exception, all participants in our cohort had a ROM of at least 50%. Considering the obtained results, we conclude that ROM alone is not a suitable measure for determining the success of neck rehabilitation outcomes. This conclusion agrees with a biopsychosocial perspective of neck pain, where pain, psychological distress, fear, self-efficacy, and physical features interact with each other and lead to disability [51]. Overall, improving cervical ROM seems to be just one of several aspects in neck pain rehabilitation.

In a meta-analysis of 40 studies, Zoete et al. [52] showed that therapies such as yoga, Pilates, tai chi, and qigong positively influenced rehabilitation success as compared to no treatment. Their findings indicate that no type of exercise is superior to others in patients with chronic, non-specific neck pain. This finding agrees with the results of our study, in which the duration of individual physiotherapy only had minor effects on rehabilitation outcome. In our sample, the cause of the complaints (surgical versus conservative medical history) was more important for patient reported outcomes than the type and duration of specific treatments.

In general, physiotherapy leads to better outcomes than no intervention in patients with neck pain, with superior results after multidisciplinary rehabilitation and multimodal exercises integrated into cognitive-behavioural therapy [53]. This claim is supported by the findings of pain expert Steven Cohen [54], who differentiates between acute pain, which is useful, protective, and has survival value, and chronic pain, which is no longer useful, can be over-protective, and might be best considered as a disease itself. In clinical practice, it is often impossible to disentangle the complex interaction between the many interacting factors that influence the patients’ subjective pain experience with neck pain [55]. We cannot say with certainty whether the nociceptive pain caused by tissue or potential tissue damage, neuropathic pain following nerve injury, nociplastic pain as a result of maladaptive changes of nociceptive processing, or mixed pain causes the patient’s discomfort. In support of our concept of multimodal therapy for neck pain, Cohen et al. [54] recommend taking a patient-centered, biopsychosocial approach for these patients by combining medication, physical exercise, and psychological support.

### Limitations

Premorbid physical condition and psychological state are critical to rehabilitation outcomes. Further differentiation of cervical spine patients, e.g., according to clinical imaging results [6], was not performed. We found that anxiety and depression are important issues for surgically treated patients with neck pain, but also for chronic pain patients in general. This association between pain, pain-related disability, and depression has been confirmed in other studies [56,57]. Therefore, we recommend integrating specific assessments in the rehabilitation of these patients that place a focus on the psychological state.

Furthermore, the retrospective design of this study prevents us from commenting on the effectiveness of specific components of the rehabilitation program, as it is impossible to account for contextual factors, natural history [58], or the regression to the mean without using a randomized interventional design that compares the rehabilitation program with a control intervention [59].

## 5. Conclusions

In chronic pain therapy, multimodal rehabilitation concepts are well established and are similar in some ways to specific neck pain therapy. Multimodal therapy of neck pain leads to comparable improvements in both conservatively treated patients and in patients who have undergone neck surgery, although we have identified specific differences between these two patient groups. High levels of disability (NDI) in patients with neck pain who were either surgically or non-surgically treated at the beginning of rehabilitation significantly predicted high levels of disability at the end of rehabilitation (NDI).

In general, patients with a higher NDI had a greater need for medical counselling and psychological intervention, but NDI scores did not significantly correlate with ROM. This lack of correlation between the range of motion and perceived disability contrasts with the relationship between PROMs and ROM in other anatomic regions. However, after adjusting for baseline differences between the two groups, the limited ROM was shown to be a predictor of perceived disability after rehabilitation.

As mentioned in the limitations, assessment of the patient’s mental status should become part of the rehabilitation routine. We conclude that the health status at baseline has a significant impact on the evaluation of success. Therefore, only a multidimensional assessment of PROMs and clinician-reported objective measures (ROM) that considers different baseline values, as in the use of the performance score presented in this paper, provides an evidence-based and person-centered approach to improving physical function and health through medical rehabilitation for patients with musculoskeletal impairments.

## Figures and Tables

**Table 1:** Sociodemographic data and baseline PRO scores.

	Total *n* = 162		Cons-Group *n* = 107		Op-Group *n* = 55			
	*n*	(%)	*n*	(%)	*n*	(%)	χ^2^	*p*-Value
Male	64	(39.5%)	41	(38.3%)	23	(41.8%)	0.186	0.66
Female	98	(60.5%)	66	(61.6%)	32	(58.1%)		
Smoker	43	(27.2%)	25	(24.3%)	18	(32.7%)	1.294	0.26
	mean	(SD)	mean	(SD)	mean	(SD)	*t*-value	*p*-value
Mean age	52.7	(8.6)	52.4	(8.2)	53.5	(9.3)	0.802	0.42
Height (cm)	170	(7.4)	170.6	(7.6)	172	(6.9)	1.424	0.16
Weight (kg)	77	(13.9)	76	(13.9)	79	(13.8)	0.819	0.42
BMI	26.4	(4.1)	26.4	(4.2)	26.5	(3.8)	0.124	0.90
ROM	58.2	(16.4)	61.6	(14.1)	51.5	(18.6)	3.895	0.001 ***
NPRS	5.1	(2.1)	5.1	(2.0)	5.1	(2.1)	0.017	0.99
EQ-5D-5L	8.3	(2.1)	8.3	(1.9)	8.5	(2.5)	0.586	0.56
mobility	1.2	(0.5)	1.3	(0.6)	1.1	(0.3)	2.20	0.029 *
self-care	1.1	(0.4)	1.0	(0.2)	1.1	(0.6)	0.86	0.39
usual activities	1.7	(0.9)	1.6	(0.9)	1.7	(0.9)	0.07	0.95
pain/discomfort	2.9	(0.8)	3.0	(0.7)	2.9	(0.8)	0.20	0.84
anxiety/depression	1.5	(0.9)	1.4	(0.6)	1.7	(1.1)	2.04	0.045 *
HAQ	0.16	(0.23)	0.15	(0.25)	0.17	(0.20)	0.505	0.62
NDI	29.1	(14.0)	28.1	(13.2)	30.9	(15.4)	1.165	0.25

Cons-group: conservative treatment; Op-group: surgical intervention; SD: standard deviation; %: percent; height in cm; weight in kg; BMI: Body Mass Index; ROM: Range of Motion in percent of normal range in the transversal plane; NPRS: Numeric Pain Rating Scale; EQ-5D-5L: European Quality of Life 5 Dimensions, 5-Level Version; HAQ: Health Assessment Questionnaire; NDI: Neck Disability Index; *** *p* < 0.001; * *p* < 0.05.

**Table 2:** Correlations of PROs and ROM score at baseline (*T*1).

	NPRS (T1)	EQ-5D-5L (T1)	HAQ (T1)	ROM (T1)
**NDI (T1)**	0.58 ***	0.74 ***	0.57 ***	−0.05
**NPRS (T1)**	-	0.52 ***	0.34 ***	−0.07
**EQ-5D-5L (T1)**	-	-	0.67 ***	−0.11
**HAQ (T1)**	-	-	-	−0.07

ROM: Range of Motion as a proportion of the normal range in the transversal plane; NPRS: Numeric Pain Rating Scale; EQ-5D-5L: European Quality of Life 5 Dimensions, 5-Level Version; HAQ: Health Assessment Questionnaire; NDI: Neck Disability Index; *** *p* < 0.001.

**Table 3:** Correlations of PROs and ROM delta scores (*T*2 − *T*1).

	NPRS (T2-T1)	EQ-5D-5L (T2-T1)	HAQ (T2-T1)	ROM (T2-T1)
**NDI (T2-T1)**	0.31 **	0.37 ***	0.24 *	−0.11
**NPRS (T2-T1)**	-	0.12	0.02	0.06
**EQ-5D-5L (T2-T1)**	-	-	0.63 ***	−0.17
**HAQ (T2-T1)**	-	-	-	−0.02

ROM: Range of Motion as a proportion of the normal range in the transversal plane; NPRS: Numeric Pain Rating Scale; EQ-5D-5L: European Quality of Life 5 Dimensions, 5-Level Version; HAQ: Health Assessment Questionnaire; NDI: Neck Disability Index; *** *p* < 0.001; ** *p* < 0.01; * *p* < 0.05.

**Table 4:** Correlations of PROMs and ROM for performance scores (T2D).

	NPRS (T2D)	EQ-5D-5L (T2D)	HAQ (T2D)	ROM (T2D)
**NDI (T2D)**	0.52 ***	0.50 ***	0.32 **	−0.33 ***
**NPRS (T2D)**	-	0.30 **	0.16	−0.12
**EQ-5D-5L (T2D)**	-	-	0.61 ***	−0.26 **
**HAQ (T2D)**	-	-	-	−0.18

ROM: Range of Motion as a proportion of the normal range in the transversal plane; NPRS: Numeric Pain Rating Scale; EQ-5D-5L: European Quality of Life 5 Dimensions, 5-Level Version; HAQ: Health Assessment Questionnaire; NDI: Neck Disability Index; *** *p* < 0.001; ** *p* < 0.01.

**Table 5:** Patient reported outcome (PRO) and range of motion (ROM) scores before and after rehabilitation treatment for total group and stratified for treatment groups.

		T1		T2			Time		Time × Group	
		Mean	(SD)	Mean	(SD)	Delta	*p*	η^2^	*p*	η^2^
NDI	Total sample	28.9	13.6	22.7	12.8	6.2	<0.001	0.24	0.050	0.03
	Cons-group	28.2	13.2	21.3	11.7	6.9	<0.001	0.29		
	OP-group	30.5	14.3	26.7	14.4	3.8	0.003	0.06		
NPRS	Total sample	5.1	2.1	3.7	1.9	1.4	<0.001	0.33	0.12	0.02
	Cons-group	5.1	2.1	3.6	1.8	1.5	<0.001	0.36		
	OP-group	5.2	2.1	4.1	1.9	1.1	<0.001	0.11		
HAQ	Total sample	0.16	0.22	0.15	0.24	0.01	0.57	<0.01	0.74	<0.01
	Cons-group	0.16	0.25	0.15	0.28	0.01	0.42	<0.01		
	OP-group	0.16	0.16	0.15	0.15	0.01	0.88	<0.01		
EQ-5D-5L	Total sample	8.3	2.1	7.4	1.8	0.9	<0.001	0.18	0.61	<0.01
	Cons-group	8.3	1.9	7.4	1.6	0.9	<0.001	0.17		
	OP-group	8.4	2.4	7.4	1.6	1.0	0.002	0.06		
EQ-5D-5L	Total sample	1.21	0.54	1.17	0.44	0.04	0.41	<0.01	0.54	<0.01
mobility	Cons-group	1.27	0.62	1.20	0.48	0.03	0.20	0.01		
	OP-group	1.10	0.30	1.12	0.32	0.02	0.90	<0.01		
EQ-5D-5L	Total sample	1.06	0.38	1.08	0.42	−0.02	0.96	<0.01	0.09	0.02
self-care	Cons-group	1.04	0.24	1.11	0.50	−0.07	0.12	0.02		
	OP-group	1.10	0.57	1.02	0.14	0.08	0.33	0.01		
EQ-5D-5L	Total sample	1.63	0.86	1.47	0.78	0.16	0.035	0.03	0.90	<0.01
usual	Cons-group	1.64	0.89	1.49	0.84	0.15	0.07	0.02		
activities	OP-group	1.60	0.80	1.42	0.64	0.18	0.18	0.01		
EQ-5D-5L	Total sample	2.94	0.77	2.33	0.70	0.61	<0.001	0.33	0.032	0.03
pain/	Cons-group	2.95	0.73	2.29	0.69	0.66	<0.001	0.38		
discomfort	OP-group	2.90	0.85	2.42	0.72	0.48	<0.001	0.09		
EQ-5D-5L	Total sample	1.49	0.83	1.30	0.57	0.19	0.007	0.05	0.98	<0.01
anxiety/	Cons-group	1.39	0.64	1.22	0.44	0.17	0.016	0.04		
depression	OP-group	1.69	1.11	1.46	0.75	0.23	0.10	0.02		
ROM (%)	Total sample	58.2	16.5	68.7	14.9	10.5	<0.001	0.58	0.13	0.02
	Cons-group	61.6	14.1	71.5	12.5	9.9	<0.001	0.47		
	OP-group	51.1	18.7	62.9	17.8	11.8	<0.001	0.38		

ROM: Range of Motion as a proportion of the normal range in the transversal plane; NPRS: Numeric Pain Rating Scale; EQ-5D-5L: European Quality of Life 5 Dimensions, 5-Level Version; HAQ: Health Assessment Questionnaire; NDI: Neck Disability Index; effect sizes: η^2^ > 0.01 = small, η^2^ > 0.06 = medium, and η^2^ > 0.14 = large effect sizes.

**Table 6:** Hierarchical multiple linear regression model for variables associated with NDI scores at the end of treatment (*T*2).

	Step 1: Sociodemographic		Step 2: Treatment		Step 3: Baseline PROMs		Step 4: Baseline NDI	
	β	*p*-Value	β	*p*-Value	β	*p*-Value	β	*p*-Value
Sex	**0.18**	**0.019**	0.08	0.32	0.06	0.37	0.03	0.66
Age	0.02	0.81	0.07	0.36	−0.10	0.16	−0.06	0.31
BMI	−0.07	0.36	−0.05	0.49	−0.06	0.35	−0.02	0.70
Smoking	−0.03	0.65	−0.07	0.33	−0.12	0.07	−0.07	0.21
Nr. medications	**0.35**	**<0.001**	**0.22**	**0.007**	0.14	0.06	0.07	0.28
Psych	-	-	**0.24**	**0.003**	0.11	0.15	0.03	0.69
Physio	-	-	0.04	0.63	0.05	0.49	0.02	0.69
Massage	-	-	−0.02	0.77	0.00	0.99	0.04	0.44
Physician	-	-	**0.21**	**0.005**	**0.14**	**0.039**	0.06	0.25
Occupational	-	-	0.06	0.39	−0.02	0.72	−0.01	0.84
EQ-5D-5L (T1)	-	-	-	-	0.14	0.13	−0.08	0.32
HAQ (T1)	-	-	-	-	**0.31**	**<0.001**	**0.14**	**0.047**
NPRS (T1)	-	-	-	-	0.11	0.13	−0.02	0.73
ROM (T1)	-	-	-	-	**−0.18**	**0.008**	**−0.17**	**0.003**
NDI (T1)	-	-	-	-	-	-	**0.66**	**<0.001**
R^2^ (Sig. model)	**0.177**	**(<0.001)**	**0.284**	**(<0.001)**	**0.480**	**(<0.001)**	**0.637**	**(<0.001)**
Δ R^2^ (Sig. of Δ R^2^)	-	-	**0.107**	**(0.001)**	**0.196**	**(<0.001)**	**0.157**	**(<0.001)**

β: standardized coefficient; nr. medications = number of medication patients are currently taking; significant predictors displayed in bold; BMI: Body Mass Index; ROM: Range of Motion as a proportion of the normal range in the transversal plane; NPRS: Numeric Pain Rating Scale; EQ-5D-5L: European Quality of Life 5 Dimensions, 5-Level Version; HAQ: Health Assessment Questionnaire; NDI: Neck Disability Index; psych = number of psychological treatment units; physio = number of physiotherapeutic treatment units; massage = number of massage treatment units; physician = number of medical consultation units; occupational = number of occupational therapy treatment units; sex: men = 0 vs. female = 1.

## Data Availability

The datasets analysed in this manuscript are not publicly available due to ethical and legal restrictions (data contain potentially identifying and sensitive patient information). If not already reported within this work, the authors may provide descriptive data on individual medical indicators for admission and discharge or the expected change due to inpatient health care for various groups and diagnoses. Requests for access to anonymized datasets should be directed to the corresponding author (vincent.grote@rehabilitation.lbg.ac.at).

## References

[1] Blanpied P.R., Gross A.R., Elliott J.M., Devaney L.L., Clewley D., Walton D.M., Sparks C., Robertson E.K. (2017). Neck Pain: Revision 2017. J. Orthop. Sports Phys. Ther..

[2] Clarke M.J., Schiefer T.K., Pichelmann M.A., Krauss W.E. (2011). Axial neck pain: A surgeon’s perspective. Clin. Pract..

[3] McLean S.M., Burton M., Bradley L., Littlewood C. (2010). Interventions for enhancing adherence with physiotherapy: A systematic review. Man. Ther..

[4] Hutting N., Mourad F., Kranenburg R., Wilbrink W., Kerry R., Taylor A. (2022). What to look out for, what to do, and when: 3 key messages for safely treating neck pain, headache and/or orofacial symptoms in musculoskeletal rehabilitation settings. J. Orthop. Sports Phys. Ther..

[5] Bowden R.E.M. (1966). Cervical Spondylosis: The Applied Anatomy of the Cervical Spine and Brachial Plexus. Proc. R. Soc. Med..

[6] Recenti M., Ricciardi C., Edmunds K., Jacob D., Gambacorta M., Gargiulo P. (2021). Testing soft tissue radiodensity parameters interplay with age and self-reported physical activity. Eur. J. Transl. Myol..

[7] Hindman B.J., Palecek J.P., Posner K.L., Traynelis V.C., Lee L.A., Sawin P.D., Tredway T.L., Todd M.M., Domino K.B. (2011). Cervical spinal cord, root, and bony spine injuries: A closed claims analysis. Anesthesiology.

[8] Yukawa Y., Kato F., Suda K., Yamagata M., Ueta T. (2012). Age-related changes in osseous anatomy, alignment, and range of motion of the cervical spine. Part I: Radiographic data from over 1200 asymptomatic subjects. Eur. Spine J..

[9] Harrop J.S., Hanna A., Silva M.T., Sharan A. (2007). Neurological manifestations of cervical spondylosis: An overview of signs, symptoms, and pathophysiology. Neurosurgery.

[10] Wong J.J., Cote P., Quesnele J.J., Stern P.J., Mior S.A. (2014). The course and prognostic factors of symptomatic cervical disc herniation with radiculopathy: A systematic review of the literature. Spine J..

[11] Zielinska N., Podgorski M., Haladaj R., Polguj M., Olewnik L. (2021). Risk Factors of Intervertebral Disc Pathology—A Point of View Formerly and Today—A Review. J. Clin. Med..

[12] Petersen J.A., Brauer C., Thygesen L.C., Flachs E.M., Lund C.B., Thomsen J.F. (2022). Prospective, population-based study of occupational movements and postures of the neck as risk factors for cervical disc herniation. BMJ Open.

[13] Rao R. (2002). Neck pain, cervical radiculopathy, and cervical myelopathy: Pathophysiology, natural history, and clinical evaluation. J. Bone Jt. Surg..

[14] Fritz J.M., Brennan G.P. (2007). Preliminary examination of a proposed treatment-based classification system for patients receiving physical therapy interventions for neck pain. Phys. Ther..

[15] Engquist M., Lofgren H., Oberg B., Holtz A., Peolsson A., Soderlund A., Vavruch L., Lind B. (2015). Factors Affecting the Outcome of Surgical Versus Nonsurgical Treatment of Cervical Radiculopathy: A Randomized, Controlled Study. Spine.

[16] Snodgrass S.J., Cleland J.A., Haskins R., Rivett D.A. (2014). The clinical utility of cervical range of motion in diagnosis, prognosis, and evaluating the effects of manipulation: A systematic review. Physiotherapy.

[17] Patrick D.L., Deyo R.A. (1989). Generic and disease-specific measures in assessing health status and quality of life. Med. Care.

[18] Brinker M.R., O’Connor D.P. (2013). Stakeholders in outcome measures: Review from a clinical perspective. Clin. Orthop. Relat. Res..

[19] Kennedy D.M., Stratford P.W., Wessel J., Gollish J.D., Penney D. (2005). Assessing stability and change of four performance measures: A longitudinal study evaluating outcome following total hip and knee arthroplasty. BMC Musculoskelet. Disord..

[20] Bily W., Jauker J., Nics H., Grote V., Pirchl M., Fischer M.J. (2022). Associations between Patient-Reported and Clinician-Reported Outcome Measures in Patients after Traumatic Injuries of the Lower Limb. Int. J. Environ. Res. Public Health.

[21] Zdravkovic A., Grote V., Pirchl M., Stockinger M., Crevenna R., Fischer M.J. (2022). Comparison of patient- and clinician-reported outcome measures in lower back rehabilitation: Introducing a new integrated performance measure (t2D). Qual. Life Res..

[22] Bruce B., Fries J. (2004). Longitudinal comparison of the Health Assessment Questionnaire (HAQ) and the Western Ontario and McMaster Universities Osteoarthritis Index (WOMAC). Arthritis Rheum..

[23] Rabin R., de Charro F. (2001). EQ-5D: A measure of health status from the EuroQol Group. Ann. Med..

[24] Vernon H., Mior S. (1991). The Neck Disability Index: A study of reliability and validity. J. Manip. Physiol. Ther..

[25] Bachner F., Bobek J., Habimana K., Ladurner J., Lepuschutz L., Ostermann H., Rainer L., Schmidt A.E., Zuba M., Quentin W. (2018). Austria: Health System Review. Health Syst. Transit..

[26] Grote V., Unger A., Bottcher E., Muntean M., Puff H., Marktl W., Mur E., Kullich W., Holasek S., Hofmann P. (2020). General and Disease-Specific Health Indicator Changes Associated with Inpatient Rehabilitation. J. Am. Med. Dir. Assoc..

[27] Grote V., Unger A., Puff H., Böttcher E., Mario B.-F., Danúbiada Cunha de S.-C., Redha T. (2020). What to Expect: Medical Quality Outcomes and Achievements of a Multidisciplinary Inpatient Musculoskeletal System Rehabilitation. Physical Therapy Effectiveness.

[28] de Koning C.H., van den Heuvel S.P., Staal J.B., Smits-Engelsman B.C., Hendriks E.J. (2008). Clinimetric evaluation of active range of motion measures in patients with non-specific neck pain: A systematic review. Eur. Spine J..

[29] Lemeunier N., Jeoun E.B., Suri M., Tuff T., Shearer H., Mior S., Wong J.J., da Silva-Oolup S., Torres P., D’Silva C. (2018). Reliability and validity of clinical tests to assess posture, pain location, and cervical spine mobility in adults with neck pain and its associated disorders: Part 4. A systematic review from the cervical assessment and diagnosis research evaluation (CADRE) collaboration. Musculoskelet. Sci. Pract..

[30] Gajdosik R.L., Bohannon R.W. (1987). Clinical measurement of range of motion. Review of goniometry emphasizing reliability and validity. Phys. Ther..

[31] Bogduk N., Mercer S. (2000). Biomechanics of the cervical spine. I: Normal kinematics. Clin. Biomech..

[32] Breivik H., Borchgrevink P.C., Allen S.M., Rosseland L.A., Romundstad L., Hals E.K., Kvarstein G., Stubhaug A. (2008). Assessment of pain. Br. J. Anaesth..

[33] Dworkin R.H., Turk D.C., Farrar J.T., Haythornthwaite J.A., Jensen M.P., Katz N.P., Kerns R.D., Stucki G., Allen R.R., Bellamy N. (2005). Core outcome measures for chronic pain clinical trials: IMMPACT recommendations. Pain.

[34] Ferreira-Valente M.A., Pais-Ribeiro J.L., Jensen M.P. (2011). Validity of four pain intensity rating scales. Pain.

[35] Fries J.F., Spitz P., Kraines R.G., Holman H.R. (1980). Measurement of patient outcome in arthritis. Arthritis Rheum..

[36] Buchholz I., Janssen M.F., Kohlmann T., Feng Y.S. (2018). A Systematic Review of Studies Comparing the Measurement Properties of the Three-Level and Five-Level Versions of the EQ-5D. Pharmacoeconomics.

[37] Devlin N.J., Brooks R. (2017). EQ-5D and the EuroQol Group: Past, Present and Future. Appl. Health Econ. Health Policy.

[38] Herdman M., Gudex C., Lloyd A., Janssen M., Kind P., Parkin D., Bonsel G., Badia X. (2011). Development and preliminary testing of the new five-level version of EQ-5D (EQ-5D-5L). Qual. Life Res..

[39] Fairbank J.C., Pynsent P.B. (2000). The Oswestry Disability Index. Spine.

[40] McCarthy M.J., Grevitt M.P., Silcocks P., Hobbs G. (2007). The reliability of the Vernon and Mior neck disability index, and its validity compared with the short form-36 health survey questionnaire. Eur. Spine J..

[41] Wagner B., Zdravkovic A., Pirchl M., Puhan M.A., Zwick R.H., Grote V., Crevenna R., Fischer M.J. (2022). Performance Score (T2D)—A New Perspective in the Assessment of Six-Minute Walking Tests in Pulmonary Rehabilitation. Diagnostics.

[42] Grote V., Pirchl M., Fischer M.J. (2021). A new perspective on stratified outcome evaluation. J. Int. Soc. Phys. Rehabil. Med..

[43] Grote V., Fischer M.J. (2022). Prospects for translational research on outcome measures in musculoskeletal rehabilitation: The search for critical success factors. Eur. J. Transl. Myol..

[44] Ellis P.D. (2012). The Essential Guide to Effect Sizes.

[45] Cha E.D.K., Lynch C.P., Jadczak C.N., Mohan S., Geoghegan C.E., Singh K. (2022). Impact of Depression Severity on Patient-Reported Outcome Measures Following Multilevel Anterior Cervical Discectomy and Fusion. Int. J. Spine Surg..

[46] Colantonio D.F., Nassr A., Freedman B.A., Elder B.D., Bydon M., Helgeson M.D., Kepler C.K., Sebastian A.S., Wagner S.C. (2022). The Effect of Preoperative Mental Health Status on Outcomes After Anterior Cervical Discectomy and Fusion. Int. J. Spine Surg..

[47] De Pauw R., Kregel J., De Blaiser C., Van Akeleyen J., Logghe T., Danneels L., Cagnie B. (2015). Identifying prognostic factors predicting outcome in patients with chronic neck pain after multimodal treatment: A retrospective study. Man. Ther..

[48] Giesinger K., Hamilton D.F., Jost B., Holzner B., Giesinger J.M. (2014). Comparative responsiveness of outcome measures for total knee arthroplasty. Osteoarthr. Cartil..

[49] Kauther M.D., Piotrowski M., Hussmann B., Lendemans S., Wedemeyer C. (2012). Cervical range of motion and strength in 4293 young male adults with chronic neck pain. Eur. Spine J..

[50] Multanen J., Hakkinen A., Kautiainen H., Ylinen J. (2021). Associations of neck muscle strength and cervical spine mobility with future neck pain and disability: A prospective 16-year study. BMC Musculoskelet. Disord..

[51] Lee H., Hubscher M., Moseley G.L., Kamper S.J., Traeger A.C., Mansell G., McAuley J.H. (2015). How does pain lead to disability? A systematic review and meta-analysis of mediation studies in people with back and neck pain. Pain.

[52] De Zoete R.M., Armfield N.R., McAuley J.H., Chen K., Sterling M. (2021). Comparative effectiveness of physical exercise interventions for chronic non-specific neck pain: A systematic review with network meta-analysis of 40 randomised controlled trials. Br. J. Sports Med..

[53] Monticone M., Cedraschi C., Ambrosini E., Rocca B., Fiorentini R., Restelli M., Gianola S., Ferrante S., Zanoli G., Moja L. (2015). Cognitive-behavioural treatment for subacute and chronic neck pain. Cochrane Database Syst. Rev..

[54] Cohen S.P., Vase L., Hooten W.M. (2021). Chronic pain: An update on burden, best practices, and new advances. Lancet.

[55] Suvinen T.I., Reade P.C., Kemppainen P., Kononen M., Dworkin S.F. (2005). Review of aetiological concepts of temporomandibular pain disorders: Towards a biopsychosocial model for integration of physical disorder factors with psychological and psychosocial illness impact factors. Eur. J. Pain.

[56] Means-Christensen A.J., Roy-Byrne P.P., Sherbourne C.D., Craske M.G., Stein M.B. (2008). Relationships among pain, anxiety, and depression in primary care. Depress. Anxiety.

[57] de Heer E.W., Gerrits M.M., Beekman A.T., Dekker J., van Marwijk H.W., de Waal M.W., Spinhoven P., Penninx B.W., van der Feltz-Cornelis C.M. (2014). The association of depression and anxiety with pain: A study from NESDA. PLoS ONE.

[58] Cashin A.G., McAuley J.H., Lamb S.E., Lee H. (2021). Disentangling contextual effects from musculoskeletal treatments. Osteoarthr. Cartil..

[59] Whitney C.W., Von Korff M. (1992). Regression to the mean in treated versus untreated chronic pain. Pain.

